# 42. METABOLISM AND CO-MORBIDITIES IN PSYCHOTIC DISORDERS

**DOI:** 10.1093/schbul/sby014.173

**Published:** 2018-04-01

**Authors:** Matej Oresic

**Affiliations:** University of Turku

## Abstract

**Overall Abstract:** Schizophrenia is associated with a reduced life expectancy of 15–20 years due to a high prevalence of cardiovascular disease and metabolic syndrome. Unhealthy lifestyles and pharmacological side effects have been suggested to be a major cause of excess mortality rates in patients with psychotic disorders. However, abnormal glucose homeostasis, hyperinsulinemia and accumulation of visceral fat are already detected in drug-naïve first episode psychosis (FEP) patients, independently of obesity.

The aim of this symposium is to present the latest research on the theme of metabolic co-morbidities in psychotic disorders. Preliminary and published data from the symposium speakers suggests that FEP is associated with altered composition of gut microbiota, inflammation and lipid dysregulation including changes in the endocannabinoid system in the central nervous system. The studies presented in this symposium may offer new insights into the gut-brain axis in psychotic disorders as well as provide new evidence on the role of lipids as a potential underlying link between the aetiology of psychosis and the associated metabolic disturbances.

All four speakers are principal investigators in the European FP7 project METSY, which aims to identify and evaluate multi-modal peripheral and neuroimaging markers that can predict and monitor psychotic and metabolic symptoms, with specific focus on metabolic co-morbidities in psychotic disorders.

Oliver Howes will first introduce the topic, based on his recent meta-analysis (JAMA Psychiatry 2017; 74; 261–269) as well as present his recent research on neuroimaging of the endocannabinoid system in FEP.

Jaana Suvisaari will present her recent research on gut microbiome and inflammation in FEP. The findings so far suggest that FEP patients have specifically altered gut microbiome composition and increased levels of inflammation.

Tuulia Hyötyläinen will introduce the field of metabolomics in psychosis research and discuss the latest findings from at-risk mental state individuals and FEP patients. Recently published findings suggest that FEP patients who later rapidly gain weight are characterised by increased markers of liver fat at the baseline.

Jarmo Hietala will present neuroimaging studies of the endocannabinoid system using PET and the [18F]FMPEP-d2 tracer, a CB1R radioligand. These studies suggest a major sex difference in the brain CB1 receptor system as well as clear evidence for a dysregulated endocannabinoid system in first-episode psychosis.

Given the endocannabinoid system in the periphery promotes the development of fatty liver, the presented work together suggests that the endocannabinoid system may be the underlying link between the psychosis and the associated metabolic disturbances. This intriguing possibility with potential diagnostic and therapeutic implications will be discussed by the presenters. The discussant Matej Oresic is the coordinator of the METSY project.

